# Impact of minimal invasive extracorporeal circulation on perioperative intravenous fluid management in coronary artery bypass surgery

**DOI:** 10.1177/02676591211043232

**Published:** 2021-09-03

**Authors:** Sten Ellam, Jenni Räsänen, Juha Hartikainen, Tuomas Selander, Auni Juutilainen, Jari Halonen

**Affiliations:** 1 Department of Anesthesiology and Operative Services, Kuopio University Hospital, Kuopio, Finland; 2 School of Medicine, University of Eastern Finland, Kuopio, Finland; 3 Heart Center, Kuopio University Hospital, Kuopio, Finland; 4 Research Support Services, Kuopio University Hospital, Kuopio, Finland

**Keywords:** minimal invasive extracorporeal circulation, MiECC, MECC, hemodilution, transfusion, packed red blood cells, intravenous fluid management, postoperative atrial fibrillation, coronary artery bypass surgery

## Abstract

**Objective:**

Compare the use of blood products and intravenous fluid management in patients scheduled for coronary artery bypass surgery and randomized to minimal invasive extracorporeal circulation (MiECC) and conventional extracorporeal circulation (CECC).

**Methods:**

A total of 240 patients who were scheduled for their first on-pump CABG, were randomized to MiECC or CECC groups. The study period was the first 84 hours after surgery. Hemoglobin <80 g/l was used as transfusion trigger.

**Results:**

Red blood cell transfusions intraoperatively were given less often in the MiECC group (23.3% vs 9.2%, p = 0.005) and the total intravenous fluid intake was significantly lower in the MiECC group (3300 ml [2950–4000] vs 4800 ml [4000–5500], p < 0.001). Hemoglobin drop also was lower in the MiECC group (35.5 ± 8.9 g/l vs 50.7 ± 9 g/l, p < 0.001) as was hemoglobin drop percent (25.3 ± 6% vs 35.3 ± 5.9%, p < 0.001). Chest tube drainage output was higher in the MiECC group (645 ml [500–917.5] vs 550 ml [412.5–750], p = 0.001). Particularly, chest tube drainage in up to 600 ml category, was in benefit of CECC group (59.1% vs 40.8%, p = 0.003). ROC curve analysis showed that patients with hemoglobin level below 95 g/l upon arrival to intensive care unit was associated with increased risk of developing postoperative atrial fibrillation (POAF) (p = 0.002, auc = 0.61, cutoff <95, sensitivity = 0.47, positive predictive value = 0.64).

**Conclusion:**

MiECC reduced the intraoperative need for RBC transfusion and intravenous fluids compared to the CECC group, also reducing hemoglobin drop compared to the CECC group in CABG surgery patients. Postoperative hemoglobin drop was a predictor of POAF.

## Introduction

A majority of the coronary artery bypass operations (CABG) are performed with the assistance of extracorporeal circulation. The most used method is the conventional extracorporeal circulation (CECC). CECC is known to be associated with undesirable side-effects such as hemodilution, increased systemic inflammatory response, and activated coagulation cascade. Hemodilution is the main reason for the use of blood products in cardiac surgery and per se is considered an independent risk factor for increased mortality and major morbidity.^1–6^

Evolution of the cardiopulmonary bypass has emerged new techniques such as minimal invasive extracorporeal circulation (MiECC). The principle behind MiECC is to reduce hemodilution and reduced blood-air and blood-artificial material contact by using centrifugal pump driven, coated, closed, and minimized circuit without open venous reservoir. Concept has theoretical benefits and evidence supporting its use.^7^

To give detailed insight to the perioperative intravenous fluid management, we report additional secondary outcomes to already published prospective, randomized, open labeled clinical study, where we compared, whether MiECC was superior to CECC in preventing postoperative atrial fibrillation after CABG surgery.^8^

## Methods

### Patients

A total of 240 patients, aged 39–82 years, scheduled to undergo their first CABG at Kuopio University Hospital between February 2010 and August 2014 were enroled. Patients with a history of episodes of atrial fibrillation or flutter, sick sinus syndrome, II-or III-degree atrioventricular block, heart failure, corticosteroid, or immunosuppressive medication, thyroid disease as well as patients, who were scheduled for redo- or emergency surgery or were enroled to another study, were excluded. Also, we excluded patients with unexpected conversion to off-pump surgery due to atheromatous ascending aorta revealed by routine perioperative epiaortic scanning.

### Study protocol

Ethics Committee of University of Eastern Finland approved the study protocol and all patients gave written informed consent. The study complies with the Declaration of Helsinki. ClinicalTrials.gov Identifier: NCT01160393.

Patients underwent on-pump elective CABG surgery with MiECC or CECC method assigned by computer-generated randomization list, study period being 84 hours.

The anesthetic management was the same in the both groups: propofol infusion, sufentanil, and pancuronium boluses. Pulmonary artery catheter was used in all patients. The mean arterial pressure target was above 60 mm Hg, and phenylephrine boluses or norepinephrine infusion were used if needed. Cardiac index target was above 2.0 l/minute/m^2^, and dobutamine infusion (2–10 μg/kg/minute) was applied when appropriate. Hemoglobin level below 80 g/l was the red blood cell transfusion trigger during the whole study period. In patients with warfarin treatment, INR value above 1.6 was the trigger for fresh frozen plasma infusion. We used Ringer acetate (Baxter) or Plasmalyte (Baxter) for crystalloid and Gelofusin (B. Braun) for colloid infusion.

We have reported detailed description of the extracorporeal circulation methods and anticoagulation protocol previously.^8^ In brief, for the MiECC method we used type III setup,^9^ with the priming volume of 1000 ml and Calafiore-type warm blood cardioplegia for the myocardial protection. Correspondingly for the CECC method we used open system setup with the priming volume of 2000 ml and Buckberg-type tepid blood cardioplegia for the myocardial protection. Components for both extracorporeal circulation methods had Softline coating. For both extracorporeal circulation methods Hepcon (Medtronic, Minneapolis, MN, USA) protocol was used, with activated coagulation time (ACT) target of 480 seconds and protamine was administered according to the Hepcon calculation, in both groups.

After surgery, the patients were followed in the intensive care unit and were weaned off ventilator when they fulfilled the following criteria: hemodynamic stability, peripheral temperature of more than 32°C, cooperativity, and no major bleeding. Patients were given per oral metoprolol of 50 mg × 3 from the first postoperative day. If the heart rate was lower than 60 beats per minute, the dose was reduced to 25 mg × 3. Chest drains were removed on the first postoperative day and patients were referred to the surgical ward.

### Outcome measures

Intraoperative outcomes: infusions (packed red blood cells (RBC)), crystalloids, colloids, fresh frozen plasma, platelets; hemoglobin, hemoglobin drop 15 minutes after onset of perfusion.

Postoperative outcomes in the intensive care unit: infusions (RBC, crystalloids, colloids, fresh frozen plasma, platelets); hemoglobin, hematocrit; time in ICU, intubation time; chest tube output; first postoperative day fluid balance and diuretics use.

In-hospital outcomes: the need of RBC during the whole study period; weight gain; correlation of the 30-minute episode of postoperative atrial fibrillation to the lowest postoperative hemoglobin level and first postoperative day fluid balance.

### Outcome parameter data

The blood sample comparison between groups was accomplished on the following time points: preoperative samples were taken 1 day before the surgery, intraoperative samples 15 minutes after the onset of the perfusion, the first intensive care unit sample shortly after arriving. The first postoperative day samples were taken in the intensive care unit on the first morning after the surgery and the three to four postoperative day samples in the ward.

The use of RBC transfusion in whole study period was divided into four categories: 0 = 0 ml, 1 = 1–500 ml, 2 = 501–1500 ml, and 3 = above 1500 ml. The volume of one RBC unit was 250 ml.

The output from the chest tubes during the first 12 hours after the surgery was measured and divided into five categories according to Universal Definition of Postoperative Bleeding (UDPB): 0 = <600 ml, 1 = 601–800 ml, 2 = 801–1000 ml, 3 = 1001–2000 ml, 4 = >2000 ml.^10^

### Statistical analysis

Continuous variables were analyzed with the Student’s *t*-test and Mann-Whitney *U* test when appropriate. Categorical variables were compared using the chi-square test or Fisher’s exact test. Normally distributed data were expressed as mean ± standard deviation (SD) and non-normal distributed data were expressed as median with interquartile range [IQR]. ROC curve analysis was used to calculate optimal cutoff value of hemoglobin to predict POAF. Multivariate logistic regression analysis was performed to assess fluid balance, as previously reported independent risk factor, linkage to POAF. IBM SPSS statistics software package version 22 was used for statistical analyses. A p-value <0.05 was considered statistically significant.

## Results

With respect to all preoperative characteristics, MiECC, and CECC groups were well matched with no significant differences between the groups (Table 1).

**Table 1.** Preoperative clinical characteristics.

	MiECC (*n* = 120)	CECC (*n* = 120)	p Value
Age (years)	65.1 ± 8.2	64.8 ± 9.3	0.791
Body surface area (m^2^)	2.0 ± 0.2	2.0 ± 0.2	0.472
Height	171.7 ± 9.5	171.2 ± 7.6	0.675
Weight	82.8 ± 16.9	81.2 ± 14.3	0.427
LVEF (%)	56.9 ± 13.4	55.6 ± 10.3	0.280
Sex (male)	98 (81.6)	97 (80.8)	0.869
COPD	13 (10.8)	6 (5.0)	0.138
Diabetes mellitus	27 (22.5)	32 (26.6)	0.123
Left main stenosis	45 (37.5)	45 (37.5)	1.000
Unstable angina pectoris	34 (28.3)	39 (32.5)	0.196
CCS/NYHA class			0.869
1	2 (1.7)	1 (0.8)	
2	35 (29.1)	26 (1.7)	
3	50 (49.6)	54 (44.5)	
4	33 (27.6)	39 (32.5)	
B-blocker	90 (75.0)	89 (74.2)	1.000
Nitroglycerin	71 (59.2)	69 (57.5)	0.896
Ca-antagonists	22 (18.3)	23 (19.2)	1.000
ACE inhibitors/ARB	63 (52.5)	74 (61.7)	0.192
Statins	117 (97.5)	110 (91.7)	0.084
Diuretics	26 (21.7)	19 (15.8)	0.321
ASA	114 (95)	114 (95)	1.000
Clopidogrel	3 (2.5)	4 (3.3)	1.000
Warfarin	3 (2.5)	2 (1.7)	1.000
Low molecular weight heparin	27 (22.5)	33 (27.5)	0.456
Hemoglobin (g/l)	143.9 ± 13.0	140.3 ± 15.7	0.051

MiECC: minimal invasive extracorporeal circulation; CECC: conventional extracorporeal circulation; ACE: angiotensin convertase enzyme; ARB: angiotensin receptor blocker; LVEF: left ventricular ejection fraction; COPD: chronic obstructive pulmonary disease; CCS: Canadian Cardiovascular Society score; NYHA: New York Heart Association classification score; SD: standard deviation.

The values are *n* (%) or mean ± SD.

### Intraoperative outcomes

**Figure 1.**
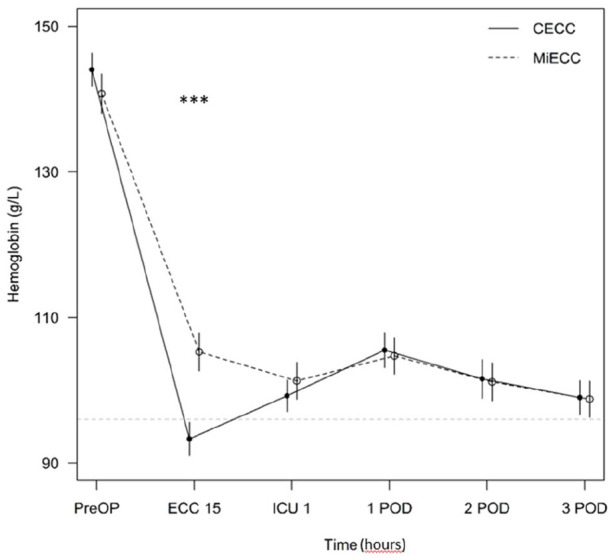
Hemoglobin drop. CECC: conventional extracorporeal circulation; MiECC: minimal invasive extracorporeal circulation; PreOP: preoperative; ECC 15: intraoperative samples 15 minutes after the onset of the perfusion; ICU 1: the first intensive care unit sample shortly after arriving; POD: postoperative day. Significance: ***p < 0.001 MiECC versus CECC.

The number of patients receiving RBC transfusion in operating theater was significantly lower in MiECC group compared to CECC group (23.3% vs 9.2%, p = 0.005, respectively) (Table 2). After the onset of perfusion, hemoglobin drop (35.5 ± 8.9 g/l vs 50.7 ± 9 g/l, p < 0.001, respectively) and hemoglobin drop percent (25.3 ± 6% vs 35.3 ± 5.9%, p < 0.001, respectively) were also significantly lower in the MiECC group compared to the CECC group (Table 2, Figure 1). In addition, total intravenous fluid intake was significantly lower in MiECC group compared to CECC group (3300 ml [2950–4000] vs 4800 ml [4000–5500], p < 0.001, respectively).

**Table 2.** Intraoperative clinical characteristics.

	MiECC (*n* = 120)	CECC (*n* = 120)	p Value
RBC transfusions given (ml)	316 [255–500]	500 [300–625]	0.125
RBC transfusions (patients)	11 (9.2)	28 (23.3)	0.005
Total IV fluid intake (ml)	3300 [2950–4000]	4800 [4000–5500]	<0.001
Hemoglobin drop (g/l)	35.5 ± 8.9	50.7 ± 9.0	<0.001
Hemoglobin drop percent (%)	25.3 ± 6	35.3 ± 5.9	<0.001
Aortic cross clamp time (minutes)	80.3 ± 25.8	77.7 ± 23.8	0.420
Perfusion time (minutes)	93.7 ± 30.4	89.5 ± 28.5	0.267

MiECC: minimal invasive extracorporeal circulation; CECC: conventional extracorporeal circulation; POAF: postoperative atrial fibrillation; iv: intravenous; [IQR]: interquartile range.

The values are *n* (%) or median (IQR) or mean ± SD.

### Postoperative outcomes in the intensive care unit

Chest tube drainage output during the first postoperative 12 hours was higher in MiECC group (645 ml [500–917.5]) compared to CECC group (550 ml [412.5–750], p = 0.001) (Table 3). The cross-tabulated categorized chest tube drainage output during the first postoperative 12 hours (UDPB) revealed statistical difference in up to 600 ml category, in benefit of CECC group compared to the MiECC group (59.1% vs 40.8%, p = 0.003, respectively) (Table 3). RBC transfusion rate, total intravenous fluid intake, ICU time, intubation time, readmission, first postoperative day fluid balance and diuretics use did not present statistical difference between study groups (Table 3).

**Table 3.** Postoperative clinical characteristics in ICU.

	MiECC (*n* = 120)	CECC (*n* = 120)	p Value
Intubation time (hours)	7.8 [6.6–10.1]	8.1 [6.5–9.9]	0.898
ICU treatment (hours)	22.6 [21.5–23.3]	22.79 [21.9–24.1]	0.207
Readmission to ICU	4/120 (3.3)	2/118 (1.7)	0.684
RBC transfusions given (ml)	500 [250–500]	375 [250–750]	0.939
RBC transfusions (patients)	27 (22.5)	26 (21.7)	1.000
Total iv fluid intake (ml)	4083 [3323–5990]	4040 [3060–5171]	0.258
One POD fluid balance	210 [–531–886]	–92 [−1176–1132]	0.154
Diuretics	48/120 (40.0)	38/120 (31.7)	0.226
Chest tube drainage UDPB (ml)			
<600	49/120 (40.8)	71/118 (60.2)	0.003
601–800	29/120 (24.2)	20/118 (16.9)	0.200
801–1000	18/120 (15.0)	9/118 (7.6)	0.101
1001–2000	22/120 (18.3)	16/118 (13.6)	0.377
>2000	2/120 (1.7)	2/118 (1.7)	1.000
Chest tube drainage (ml)	645 [500–917.5]	550 [412.5–750]	0.001
Resternotomy	4 (3.3)	7 (5.8)	0.539

MiECC: minimal invasive extracorporeal circulation; CECC: conventional extracorporeal circulation; RBC: red blood cells; iv: intravenous; POD: postoperative day; UDPB: universal definition of postoperative bleeding; [IQR]: interquartile range.

The values are mean ± standard deviation, *n* (%) or median [IQR].

### In-hospital outcomes

RBCs was given in 33 patients for the MiECC group and 44 patients in the CECC group. The cross-tabulated categorized transfusion amount of RBC did not reveal statistically significant difference between the study groups (Table 4). Perioperative body weight dynamics had no statistical difference between the study groups. The use of colloids (gelatin), fresh frozen plasma, platelets were rare and did not present statistical differences between the study groups. No patients developed acute kidney failures requiring hemodialysis.

**Table 4.** In-hospital clinical characteristics.

	MiECC (*n* = 120)	CECC (*n* = 120)	p Value
Non-transfused	87/120 (72.5)	76/120 (63.3)	0.167
RBC 0–500 ml	21/120 (17.5)	24/120 (20.0)	0.741
RBC 501–1500 ml	12/120 (10.0)	18/120 (15.0)	0.329
RBC >1500 ml	0/120 (0.0)	2/120 (1.7)	0.498
Weight 2 POD	86.6 ± 16.6	86.3 ± 14.2	0.869
Resternotomy	4 (3.3)	7 (5.8)	0.539

MiECC: minimal invasive extracorporeal circulation; CECC: conventional extracorporeal circulation; RBC: red blood cells; POD: postoperative day.

The values are mean ± standard deviation, *n* (%) or median [IQR].

Including all patients in the analysis, we found a significant correlation between the now-onset postoperative atrial fibrillation (POAF) and first hemoglobin level, measured shortly after ICU admission. ROC curve analysis showed that patients with hemoglobin level below 95 g/l, presented increased risk of developing POAF (p = 0.002, auc = 0.61, cutoff <95, sensitivity = 0.47, specificity = 0.73, and positive predictive value 0.64). However, in the multivariate logistic regression model, the first postoperative day fluid balance was not an independent predictor of POAF (OR 1.00 per 100-ml increase; 95% CI 0.99–1.02, p = 0.692).

## Discussion

The main finding of our study was that MiECC was associated with less need for red blood cell transfusions in the operating room and lower hemoglobin drop in patients undergoing CABG. In addition, hemoglobin drop upon arrival to intensive care unit was a significant predictor of POAF.

The use of allogenic blood products has been shown to be an independent predictor of morbidity and mortality in patients undergoing cardiac surgery. Even transfusions of 1–2 units of RBC, have been connected to higher morbidity, such as prolonged ventilator support, renal failure, stroke, myocardial ischemia and infections, as well as increased early and late mortality.^1–3^ The deleterious effects of red blood cell transfusions are caused by immunomodulation, transfusion of cytokines, acute lung injury, transmission of bacterial infection and increased permeability of pulmonary vasculature.^1^ The need of red blood cell transfusions is mostly related to perioperative bleeding and hemodilution, both of them are linked with increased risk of stroke after cardiac surgery.^11^ MiECC is advanced method, developed to reduce the risks related to CECC. Indeed, in our study the need of red blood cell transfusions intraoperatively was 21% lower in the MiECC group as compared to the CECC group. However, our study fell short to show any overall reduction for the need of RBC in the MiECC group. This in contrast with our earlier retrospective study of 1248 patients^12^ as well as the contemporary studies from Anastasiadis et al.,^13^ Harling et al.,^14^ Zangrillo et al.,^15^ van Boven et al.,^16^ and Benedetto et al.^17^ together with the 500-patient randomized trial from El-Essawi et al.^18^ reporting that MiECC was associated with reduction in the need of RBC transfusions. MiECC is also integrated in 2019 EACTS/EACTA/EBCP guidelines on cardiopulmonary bypass in adult cardiac surgery for avoiding hemodilution, blood loss, and maintenance of hemostasis.^19^

Allogenic blood product transfusions are mostly used in patients with blood loss and hemodilution, which per se is a common phenomenon in cardiac surgery with extracorporeal circulation. Moderate hemodilution can provide some advantages such as increased regional blood flow, decreased vascular resistance and blood viscosity. We pinpointed that, intraoperatively there was significantly increased use of intravenous fluids in the CECC group, compared to MiECC group, contributing to the excess hemodilution. Karkouti et al.,^4^ Habib et al.,^6^ and Ranucci et al.^20^ have previously demonstrated that severe hemodilution is an independent risk factor for stroke, myocardial infarction, stroke, low output syndrome, renal failure, and multiorgan failure during cardiopulmonary bypass also in patients who do not get blood transfusions. Also, co-morbidities affecting microvascular circulation seem to worsen the adverse effects caused by hemodilution during extracorporeal circulation.^21^ In our study hemodilution, assessed as hemoglobin drop, was significantly less (28%) in patients undergoing MiECC as compared to those undergoing CECC. In this respect, our results are in line with earlier registry studies and randomized controlled trials.^22–26^

We were able to demonstrate a significant difference in the 12-hour overall chest drainage output in favor of CECC operated patients. A significant difference was also present in up to 600 ml group according UDPB classification. This is in contrast to Sakwa et al.^24^ who reported c. 50% reduction of chest tube output in patients in the MiECC circuit group. However, excess bleeding in MiECC group was not mirrored to postoperative RBC transfusions or total intravenous fluid infusion amounts in the ICU or need for resternotomy. For that reason, we consider chest tube drainage difference clinically irrelevant.

ROC curve analysis showed that, in our study, patients with hemoglobin level below 95 g/l, presented increased risk of developing POAF. Using postoperative hemoglobin 95 g/l as cut-off identified 47% of patients who develop POAF. Correspondingly, of patients with hemoglobin <95 g/l 64% developed POAF. Thus, hemoglobin below 95 g/l almost doubled the risk of POAF. Lim et al.^27^ in their recent, largest ever, nationwide population-based study of 9.6 million people concentrating on the relations of hemoglobin level and atrial fibrillation, reported increased risk of atrial fibrillation in anemic patients. Sudden hemoglobin level drop and hemodilution after onset of extracorporeal circulation (below 120 g/l in female and 130 g/l in male^27^) could explain POAF in 35% of the patients in both our study groups.^8^ Hosokawa et al.^28^ in their prospective study of almost 300 off-pump CABG patients and Kalus et al.^29^ demonstrated that postoperative fluid balance is an independent predictor of POAF. In contrast, we could not present linkage between first postoperative day fluid balance and POAF.

In our study, the postoperative follow-up was 84 hours. According to our hospital practice, patients with uncomplicated recovery are discharged back to the referral hospital on the fourth postoperative day. Therefore, we do not have data on the use of blood products or fluid balance beyond the first 84 postoperative hours. However, blood products and fluids are typically given during the intraoperative period, intensive care treatment, and the first postoperative days. These periods were well covered in our study.

The need of RBC during the whole study period was 33% higher in the CECC than in the MiECC group. However, the difference did not reach statistical significance. Most probably, our study was underpowered to demonstrate the difference. In our earlier, non-randomized retrospective study with more than 1200 patients, MiECC was associated with the lower need of RBC.^12^

## Conclusion

MiECC reduced the intraoperative need for RBC transfusion and intravenous fluids compared to the CECC group. MiECC also reduced hemoglobin drop compared to the CECC group. Our findings are in line with the contemporary MiECC practice reported in the literature.

Anemia seems to be an independent risk factor for developing POAF after CABG surgery, creating a field for further high-quality studies.
